# Self-reported musculoskeletal complaints and injuries and exposure of physical workload in Swedish soldiers serving in Afghanistan

**DOI:** 10.1371/journal.pone.0195548

**Published:** 2018-04-05

**Authors:** Alexandra Halvarsson, Ingela Hagman, Matthias Tegern, Lisbet Broman, Helena Larsson

**Affiliations:** 1 Karolinska Institutet, Department of Neurobiology, Care Sciences and Society, Division of Physiotherapy, SE, Huddinge, Sweden; 2 Allied Health Professionals Function, Karolinska University Hospital, Stockholm, Sweden; 3 Department of Community Medicine and Rehabilitation, Physiotherapy, Umeå University,Umeå, Sweden; 4 Swedish Armed Forces, Headquarters, Medical Services, Stockholm, Sweden; University of Illinois at Urbana-Champaign, UNITED STATES

## Abstract

**Background:**

Musculoskeletal complaints and injuries (MSCI) are common in military populations. However, only a limited number of studies have followed soldiers during international deployments and investigated the prevalence of MSCI during and at the end of their deployment. The aim was to describe the prevalence of MSCI in different military occupational specialties and categorise their most common tasks in terms of exposures to physical workloads during a six-month long international deployment in Afghanistan.

**Methods:**

Cross-sectional survey, including 325 soldiers (300 men), aged 20–62 participating in an international deployment in Afghanistan during the spring of 2012. Soldiers were clustered into different military occupational specialties: Infantry, Administration, Logistics, Logistics/Camp, Medical and Other. Data were collected through the use of the Musculoskeletal Screening Protocol at the end of the international mission.

**Results:**

Forty-seven percent reported MSCI during deployment, with 28% at the end.

The most common locations of MSCI during the mission were lower back, knee, shoulders, upper back, neck and foot, while the knee and lower back prevailed at the end of the mission. Almost half of the soldiers who had MSCI reported affected work ability. The most common duties during the mission were vehicle patrolling, staff duties, guard/security duties, foot patrols and transportation. Soldiers reported that vehicle patrolling, staff duties and transportation were demanding with respect to endurance strength, guard/security duties challenged both maximum and endurance strength while foot patrolling challenged maximum and endurance strength, aerobic and anaerobic endurance and speed.

**Conclusions:**

MSCI during international deployment are common among Swedish soldiers. The results indicate the need to further develop strategies focusing on matching the soldiers’ capacity to the job requirements, with relevant and fair physical selection-tests during the recruitment process and proactive interventions targeting MSCI before and during deployment, in order to enhance soldiers’ readiness and promote operational readiness.

## Introduction

Musculoskeletal injuries are common in military populations [1, 2, 3, 4, 5]. During military basic training, 28–61% of Nordic soldiers report musculoskeletal disorders or injuries [6, 7, 8]. Some injuries are caused by acute traumas, but overuse is the most common reason [3, 5, 7]. According to Hauret et al, [5], overtraining, repetitive movements, forceful actions, vibrations and time spent in static positions are among other activities related to overuse injuries. Furthermore, in a study of US soldiers serving in Afghanistan, the most common causes of musculoskeletal injuries were overuse, exacerbations of previous injuries, weight lifting, sports and foot patrolling in uneven terrain [9]. Other factors that could be associated with musculoskeletal injuries are older age, female gender, number of months deployed, prolonged standing, and load carrying or lifting [10].

The external load of the equipment soldiers carry may exceed the tissues’ physiological tolerance level and thereby lead to load-related musculoskeletal complaints and injuries. On-going complaints and injuries have also been shown to affect the work ability of soldiers and may lead to a chronic condition, i.e. chronic pain, in the long term [11]. In a Swedish report, it is documented that Swedish rifle soldiers are exposed to 39 kg of combat gear, machine gun soldiers carry 49.6 kg, and radio operators 60.5 kg. The combat gear includes uniform, boots, gloves, ballistic eyewear, helmet, night vision goggles, body armour system, weapons, ammunition, tactical load vest, first aid kit, but no rucksack. Goff et al. reported similar combat gear in US soldiers, with a weight of 46.3 kg. The aforementioned authors stated that carried loads approximating the weight of the body, for prolonged periods and at repeated deployments, result in overuse injuries and chronic pain [11]. Glad et al. found that 70% of the Swedish Armed Forces (SwAF) personnel serving in Afghanistan reported musculoskeletal disorders, of which 58% occurred during deployment. The majority of the disorders were of mild character, with low pain/low disability which did not influence daily duties. [12] However, it is important to revisit these issues, since studies have found that both the working load and work rate has increased among soldiers in recent years, which may lead to increased likelihood of deteriorating health [13, 14]. In addition to the negative effects of physical workloads on soldiers’ health, psychological demands are also heightened during international deployment due to prolonged periods of staying mentally prepared for events such as suddenly launched missions, and a great variety of tasks, which may lead to uncertainty and stress. Also, the mental environment can be stressful, mainly relating to the experience of being in a war zone [13]. Soldiers work in a performance-based environment [13], and experiencing injuries, specifically reducing exercise capacity and working ability in these conditions will thereby reduce their military readiness [3].

The aim of this study was to describe the prevalence of musculoskeletal complaints and injuries in different Swedish military occupational specialties, and categorise their most common tasks in terms of exposures of physical workloads during a six-month long international deployment in Afghanistan.

## Material and methods

A descriptive, cross-sectional design was adopted to answer the study objectives. The overall inclusion criterion was that soldiers had to be deployed in an operational unit during 2012 (n = 378).

To ensure a representative selection, soldiers were recruited from different sub-units within the operational unit (staff, rifle companies, maintenance and supply). Fifty-three soldiers declined participation in the study. During the summer and autumn of 2011, soldiers received written information about the study, and at the end of their international deployment in the spring of 2012 they received repeated oral and written information at the military camp in Mazar-e-Sharif in Afghanistan. Subsequently, they were asked to provide written informed consent. The study was approved by the Ethical Committee in Stockholm, Dnr. 2011/928-32.

### Measurement

Data were collected using the Musculoskeletal Screening Protocol (MSP) questionnaire [8]. Questions included in the MSP were:

- Occurrence of musculoskeletal complaints and injuries (MSCI) before, during and at the end of deployment, with possible response options “yes” or “no”;
- Anatomical location of MSCI (neck, upper back, lower back, shoulders, elbow, hand, hip, knee, lower leg, foot) during and at the end of deployment with response options “yes” or “no”
- Frequency of MSCI during deployment with response options “seldom”/“often”/“all the time”
- To what extent musculoskeletal complaints and injuries has affected daily duties, with response options “not at all”/“to some extent”/“to quite a great extent”/“to a great extent”;
- Consultation with a medical doctor, nurse or physiotherapist with response options “yes” or “no”
- Frequency of performed muscle strength training and cardiovascular training during deployment with an open response option (sessions/week)
- Physical and mental preparedness before the mission with response options “yes” or “no”
- Most common tasks during deployment with an open answer option;
- Estimation of the exposure of physical workload for most common tasks, i.e. weight of uniform/equipment carried (open answers in “kg” and answers were grouped into <10 kg, 10.1–30 kg, 30.1–40 kg and >40 kg), duration (open answers, “hours a day”), frequency (“occasionally”/“1–2 times per week”/“3–4 times per week”/“every day of the week”).
- Estimation of the longest walking distance with open answer option (km). The answers were grouped into <10 km, 10–20 km, 20.1–30 km, 30.1–40 km and >40 km.
- Estimation of heaviest lift during deployment with open answer option (kg).
- Five questions regarding perceived self-rated health, i.e. how do you perceive your physical body, mental health, social environment, physical environment and work ability, respectively. The answers are grouped into bad, good and excellent. [15]

### Statistical analyses

For statistical calculations, IBM SPSS Statistics version 22 was used. Descriptive data are presented as number of (n), mean, standard deviation (SD), frequency (min-max), percentage (%) and 95% confidence interval (95% CI).

## Results

A total of 325 soldiers, representing different military occupations, were included in the study. Soldiers were clustered into different Military Occupational Specialties (MOS): Infantry (n = 120), Logistics (n = 50), Logistics/camp (n = 59), Medical, consisting of the medical evacuation team and medical personnel working at the camp’s health centre (n = 18), Administration (n = 49) and Other (n = 29). The group ‘Other’ consisted of soldiers with mentoring assignments (i.e. mentoring of Afghan Police and Afghan Army) and liaison (including duties both at camp and in the field, where they wear only uniform as well as combat gear). For demographic data, see Table 1.

**Table 1:** Demographic data of the included 325 soldiers clustered into different military occupational specialties, i.e. Infantry, Logistics, Logistics/camp, Medical, Administration and Other.

	n	gender (male/female), n	age, mean (SD)
Total	325	300/25	30 (9)*
Infantry	120	119/1	25 (3)
Logistics	50	47/3	27 (6)
Logistics/camp	59	57/2	33 (11)
Medical	18	11/7	38 (7)
Administration	49	39/10	37 (11)
Other	29	27/2	33 (10)*

* one missing value

Twenty-six percent of the soldiers reported having experienced a musculoskeletal complaint or injury before deployment, and out of these, 57% indicated that their complaints or injuries had deteriorated during deployment, while 43% reported improvements during deployment. The most frequent location of MSCI before deployment was the knee (8%) and lower back (5%).

**Fig 1:**
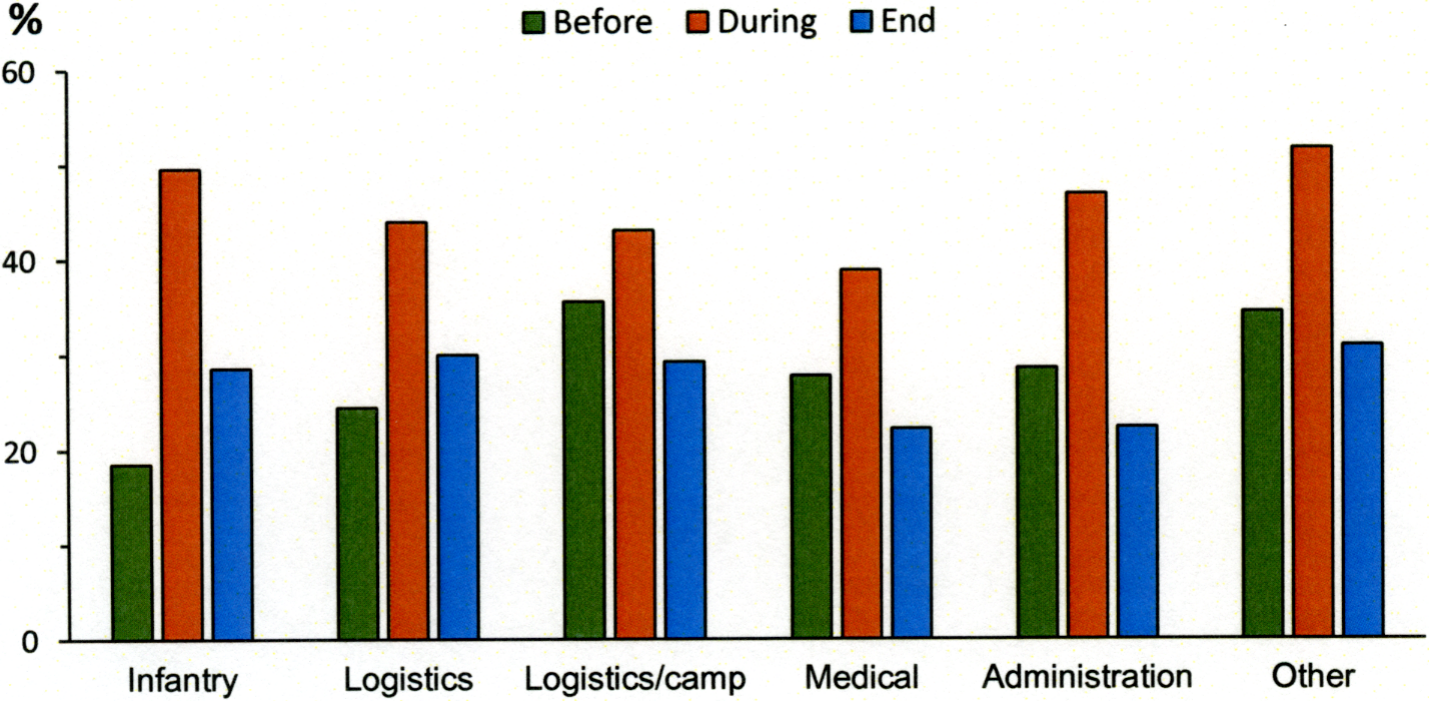
Prevalence of musculoskeletal complaints or injures of any body part for each military occupational specialty before, during and at the end of deployment.

**Fig 2:**
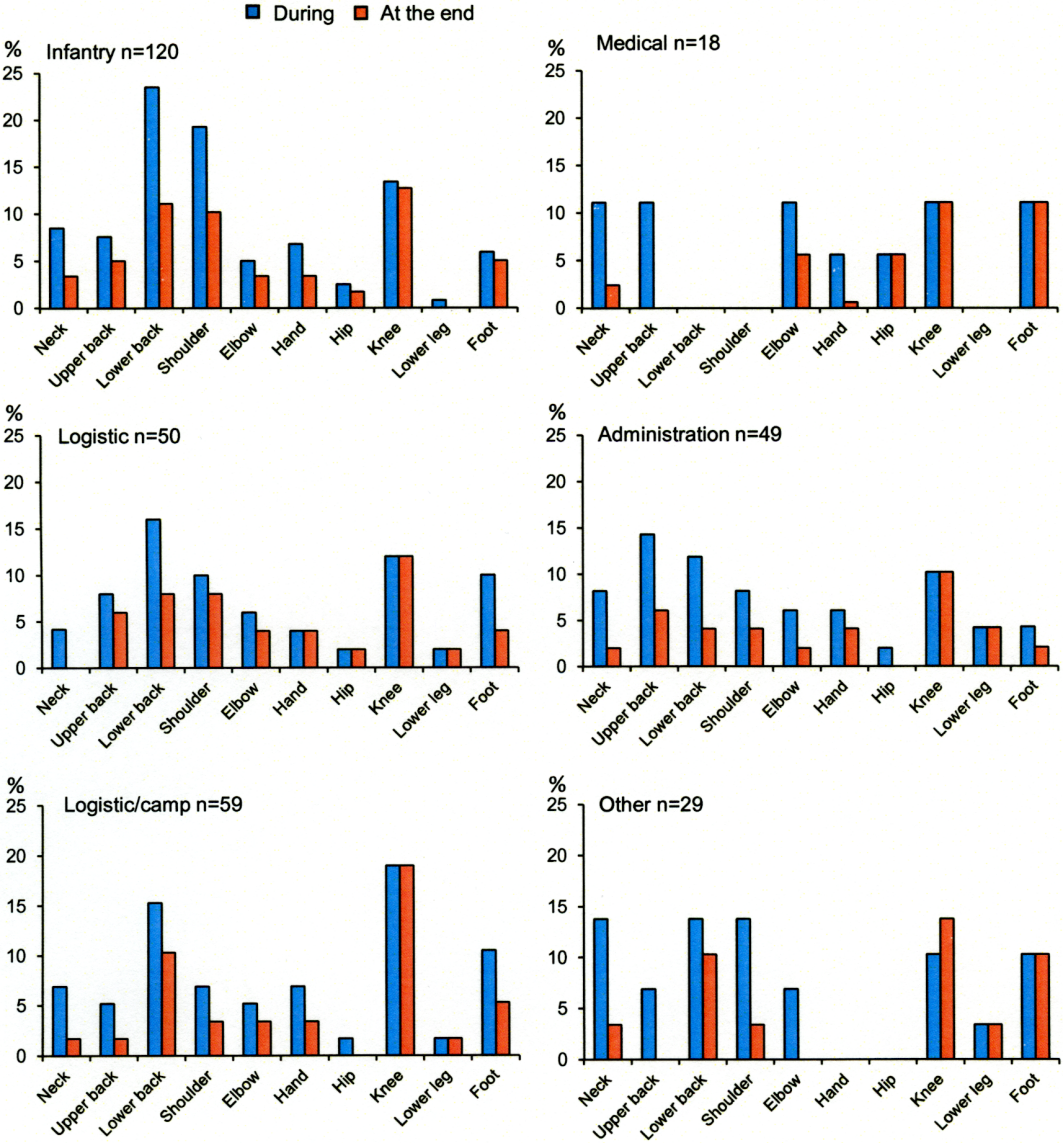
Anatomic location of musculoskeletal complaint or injuries during and at the end of deployment for each military occupational specialty.

Musculoskeletal complaints or injuries were reported in 47% of soldiers during deployment and 28% at the end of deployment. See Fig 1 for prevalence of musculoskeletal complaints or injures in each military occupational specialties before, during and at the end of deployment. Twenty-eight percent reported different MSCI both during and at the end of deployment. Furthermore, soldiers reported up to five different locations of MSCI during deployment and up to four at the end of deployment. See Fig 2 for location of MSCI for each military occupational specialty.

The frequency of MSCI was reported to be “often to always” in 22% of all soldiers in this cohort. For the different military occupational specialties, the frequency of experiencing musculoskeletal complaints and injuries “often to always” was: Infantry 27%, Logistics 9%, Logistics/Camp 26%, Medical 14%, Administration 22% and Other 13%. Almost half (53%) of the soldiers who had MSCI reported their work ability to be affected by it. Few of them rated that the impact of MSCI on work ability was to a great extent (4%). Five percent (n = 7) of the soldiers were relieved of their duties during deployment due to their MSCI. Approximately one third consulted a medical doctor, nurse or physiotherapist.

Physical training during deployment was performed by 98.5% of all soldiers, while the median of muscle strength training sessions and cardiovascular training sessions was 3 times/week and 2 times/week respectively. Almost all soldiers reported that they were motivated to undertake the deployment (98%), and 99.7% reported that they felt psychologically prepared before deployment. Four percent of the soldiers perceived that their physical performance was not sufficient for their occupational duties; most representatives were in the military occupational specialty Infantry (7%) and Medical (6%). They reported that both their muscle strength and aerobic fitness, as part of physical characteristics, were insufficient for their respective duties.

### Common task

The most common tasks performed in the whole group were vehicle patrolling (47%), administrative/staff duties (33%), guard/security duties (22%), foot patrolling (15%), transport (15%) and maintenance of vehicles and equipment care (11%). See Table 2 for the most common tasks across the different military occupational specialties.

**Table 2:** Frequency of the most common tasks in each military occupational specialty (Infantry, Logistic, Logistics/Camp, Medical, Administration and Other).

Infantry		Logistic		Logistic/camp		Medical		Administration		Other	
n = 118	%	n = 48	%	n = 57	%	n = 18	%	n = 46	%	n = 28	%
	95% CI		95% CI		95% CI		95% CI		95% CI		95% CI
Vehicle	80.5	Transport	54.2	Admin.	61.4	Medical	94.4	Admin.	100.0	Mentor	46.4
patrolling	72.5–86.7		40.3–67.4	duties	48.4–72.9	readiness	74.2–99.0	duties	92.3–100		29.5–64.2
Guard/	44.9	Vehicle	35.4	Vehicle and	24.6	Material	22.2	Vehicle	28.3	Vehicle	42.9
security	36.3–53.9	patrolling	23.4–49.6	equipment care	15.2–37.1	service	9.0–45.2	patrolling	17.3–42.6	patrolling	26.5–60.9
Foot	35.6	Medical	16.7	Technical	21.1	Vehicle	16.7	Transport	15.2	Admin.	39.3
patrolling	27.5–44.6	readiness	8.7–29.6	services	12.5–33.3	patrolling	5.8–39.2		7.6–28.2	duties	23.6–57.6
Vehicle and	21.2	Vehicle and	16.7	Supply	19.3	Guard/	11.1	Liaison	13.0	Transport	17.9
equipment care	14.8–29.4	equipment care	8.7–29.6	services	11.1–31.3	security	3.1–32.8		6.1–25.7		7.9–35.6
Material	8.5	Guard/	14.6	Transport	14.0	Foot	5.6	Training/	6.5	Close	14.3
service	4.7–14.9	security	7.3–27.2		7.3–25.3	patrolling	1.0–25.8	education	2.2–17.5	protection	5.7–31.5
Admin.	5.9	Admin.	12.5	Vehicle	12.3	Staff	5.6	Guard/	4.3	Liaison	10.7
duties	2.9–11.7	duties	5.9–24.7	patrolling	6.1–23.3	duties	1.0–25.8	security	1.2–14.5		3.7–27.2
Staff	5.9	EOD	10.4	Material	8.8			Supply	2.2	Guard/	10.7
duties	2.9–11.7		4.5–22.2	service	3.8–18.9			services	0.4–11.3	security	3.7–27.2
EOD	3.4	Recovery	8.3	Staff	8.8			Material	2.2	Training/	7.1
	1.3–8.4		3.3–19.6	duties	3.8–18.9			service	0.4–11.3	education	2.0–22.6
Training/	2.5	Repair of vehicles	8.3	Training/	5.3			Medical	2.2	Material	3.6
education	0.9–7.2	and equipment	3.3–19.6	education	1.8–14.4			readiness	0.4–11.3	service	0.6–17.7
Liaison	1.7	Material	6.3	Recovery	3.5			Foot	2.2	Vehicle and	3.6
	0.5–6.0	service	2.2–16.8		1.0–11.9			patrolling	0.4–11.3	equipment care	0.6–17.7
Medical	0.8	Foot	6.3	Other	3.5					Military police	3.6
readiness	0.2–4.7	patrolling	2.2–16.8		1.0–11.9					in the field	0.6–17.7
		Supply	4.2	Guard/	1.8						
		services	1.2–14.0	security	0.3–9.3						
		Technical	4.2	Medical	1.8						
		services	1.2–14.0	readiness	0.3–9.3						
		Liaison	2.1								
			0.4–10.9								
		Training/	2.1								
		education	0.4–10.9								
		Staff	2.1								
		duties	0.4–10.9								

EOD: Explosive Ordnance Disposal. Admin. Duties: Administrative duties

### Physical workload and estimated exposures

**Fig 3:**
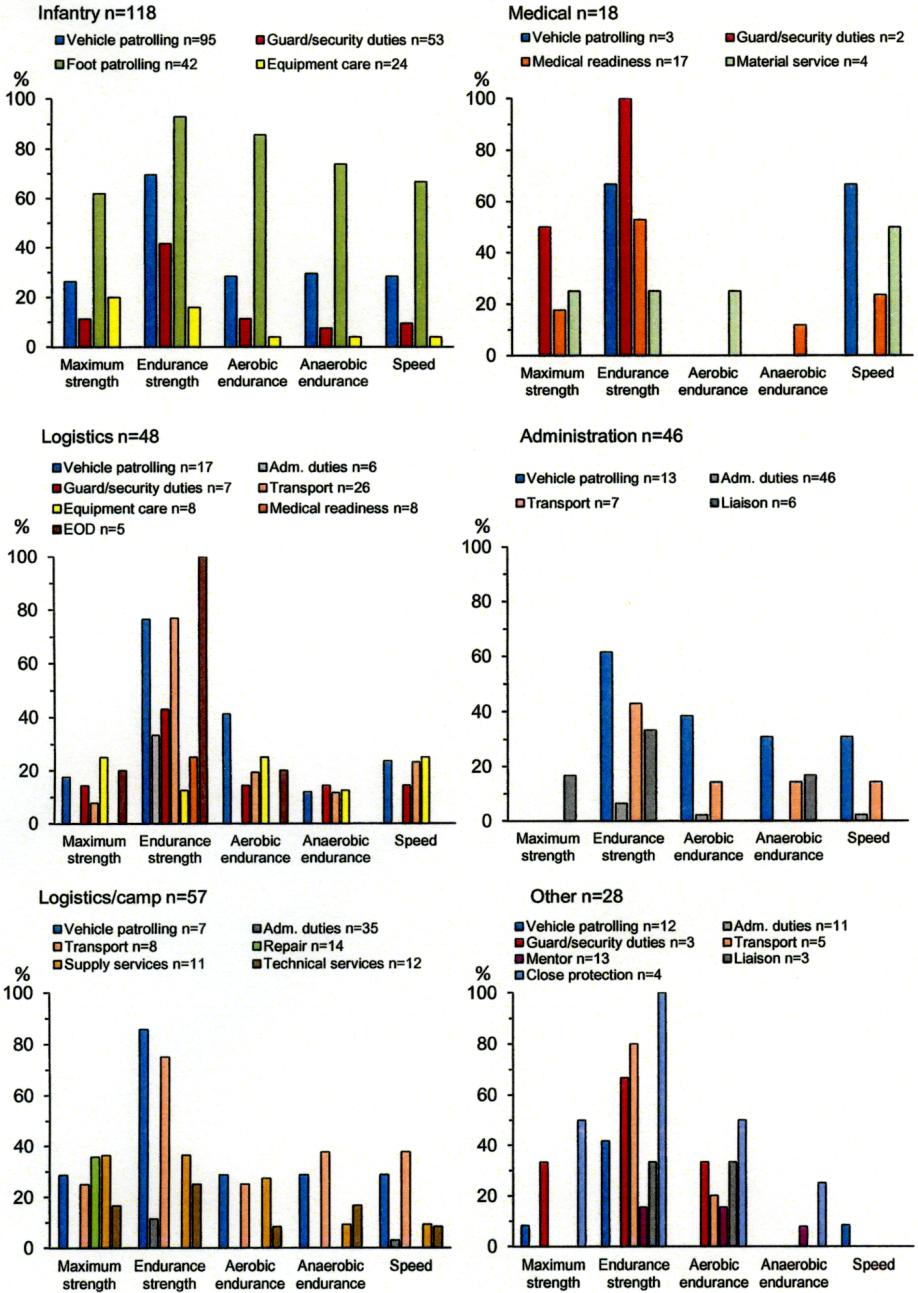
Frequency of estimated physical workload and estimated exposures (maximum strength, endurance strength, aerobic endurance, anaerobic endurance and speed) for the most commonly executed tasks across the different military occupational specialties.

**Fig 4:**
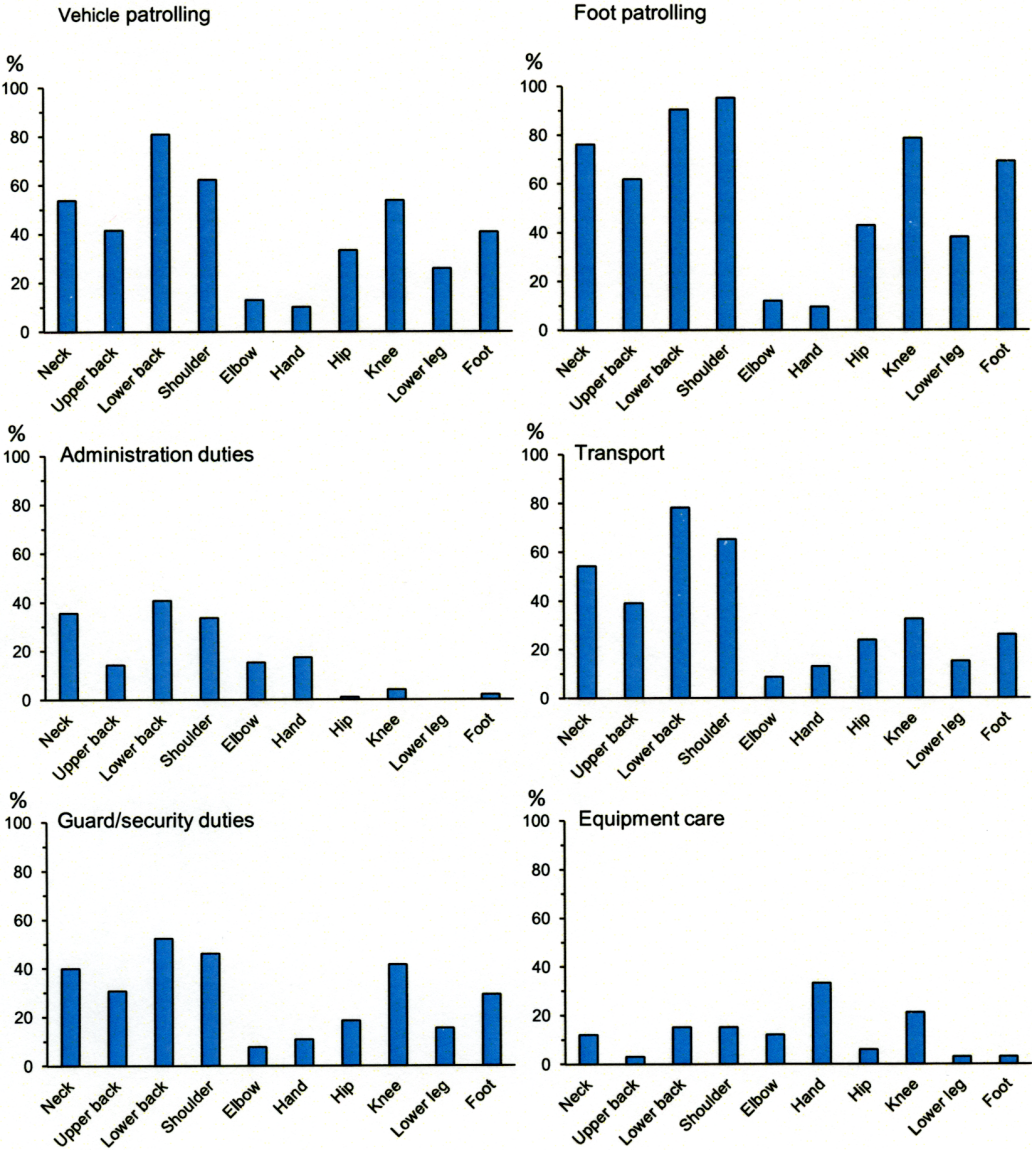
Frequency of estimation of anatomical body parts that are mainly loaded during the most commonly executed tasks across the different military occupational specialties.

Muscle endurance was the commonest estimated physical exposure for several of the working tasks reported by soldiers across the different military occupational specialties during the international mission, Fig 3. Also, the lower back and shoulders were considered the most commonly loaded anatomical body part for five of the six most common working tasks, followed by knee and neck, Fig 4.

### Exposure of physical workload during the most common tasks

For soldiers in the infantry, vehicle patrolling was the most commonly performed tasks, and 67% reported that they wore equipment with a weight of 30.1–40 kg 3–4 times/week, lasting from a few hours daily up to over seventeen hours. In MOS Logistics, transportation was the most commonly performed task. Soldiers reported that they wore equipment with a weight of 30.1–40 kg during transportation between 3–4 times/week, lasting from a few hours up to eleven hours. For soldiers in Logistics/Camp, the most common task was administrative/staff duties, where they wore equipment with a weight of 6–10 kg daily for up to six hours.

In MOS Medical Evacuation team, the most common task was medical readiness, and soldiers in this specialty wore equipment with a weight of 30.1–40 kg between 1–2 times/week to 3–4 times/week lasting a few hours up to seventeen hours. For soldiers in Administration, the most common task was administrative/staff duties, where they wore equipment with a weight of 6–10 kg, lasting, daily, from a few hours up to over seventeen hours. In MOS Other, the most common task was mentoring, and the weight of their equipment ranged from 6–10 kg daily, lasting from a few hours up to seventeen hours.

Forty-four percent of all soldiers reported that the longest distances they had walked were below 10 kilometres, while only 4% of them had walked over 30 kilometres. The most common weight of equipment that they have carried during their longest walking distances was 10.1–30 kg. See Tables 3 and 4 for more detailed information regarding longest walking distance and weight of carried equipment across all military occupational specialties.

**Table 3:** Longest reported walking distance for each military occupational specialty.

		<10 km	10–20 km	20.1–30 km	30.1–40 km	>40 km
		%	%	%	%	%
	n	95% CI	95% CI	95% CI	95% CI	95% CI
Infantry	108	28.7	59.3	5.5	4.6	1.9
		21.0–37.9	49.8–68.1	2.6–11.6	2.0–10.4	0.5–6.5
Logistics	46	65.2	32.6	0.0	2.2	0.0
		50.8–77.3	20.9–47.0	0.0–7.7	0.4–11.3	0.0–7.7
Logistics/Camp	53	41.5	30.2	22.6	1.9	3.8
		29.3–54.9	19.5–43.5	13.5–35.5	0.3–10.0	1.0–12.8
Medical	16	81.3	18.7	0.0	0.0	0.0
		57.0–93.4	6.6–43.0	0.0–19.4	0.0–19.4	0.0–19.4
Administration	43	48.9	46.5	2.3	0.0	2.3
		34.6–63.3	32.5–61.1	0.4–12.1	0.0–8.2	0.4–12.1
Other	25	48.0	48.0	4.0	0.0	0.0
		30.0–66.5	30.0–66.5	0.7–19.5	0.0–13.3	0.0–13.3

**Table 4:** Weight of carried equipment during their longest walking distance across military occupational specialties.

		0–5.9kg	6–10kg	10.1–30kg	30.1–40kg	>40kg
		%	%	%	%	%
	n	95% CI	95% CI	95% CI	95% CI	
Infantry	113	4.4	0.9	32.7	44.2	17.8
		1.9–9.9	0.2–4.8	24.8–41.8	35.4–53.5	11.8–25.8
Logistics	44	2.3	6.8	56.8	31.8	2.3
		0.4–11.8	2.4–18.2	42.2–70.3	20.0–46.6	0.4–11.8
Logistics/Camp	47	34.0	12.8	31.9	14.9	6.4
		22.2–48.3	6.0–25.2	20.4–46.2	7.4–27.7	2.2–17.2
Medical	16	18.7	6.3	43.7	31.3	0.0
		6.6–43.0	1.1–28.3	23.1–66.8	14.2–55.6	0.0–19.4
Administration	34	47.1	2.9	26.5	17.6	5.9
		31.5–63.3	0.5–14.9	14.6–43.1	8.4–33.5	1.6–19.1
Other	25	36.0	0.0	24.0	32.0	8.0
		20.3–55.5	0.0–13.3	11.5–43.4	17.2–51.6	2.2–25.0

Seventy-eight percent of the soldiers answered a question regarding the heaviest lift, i.e. >110kg (242 lbs) during deployment, and 59 soldiers reported that they lifted this amount during training sessions in the gym, while 14 others reported having lifted that amount during their occupational duties. This was reported by soldiers from several military occupational specialties, i.e. Administration, Logistics, Logistics/Camp and Infantry.

### Perceived health

**Fig 5:**
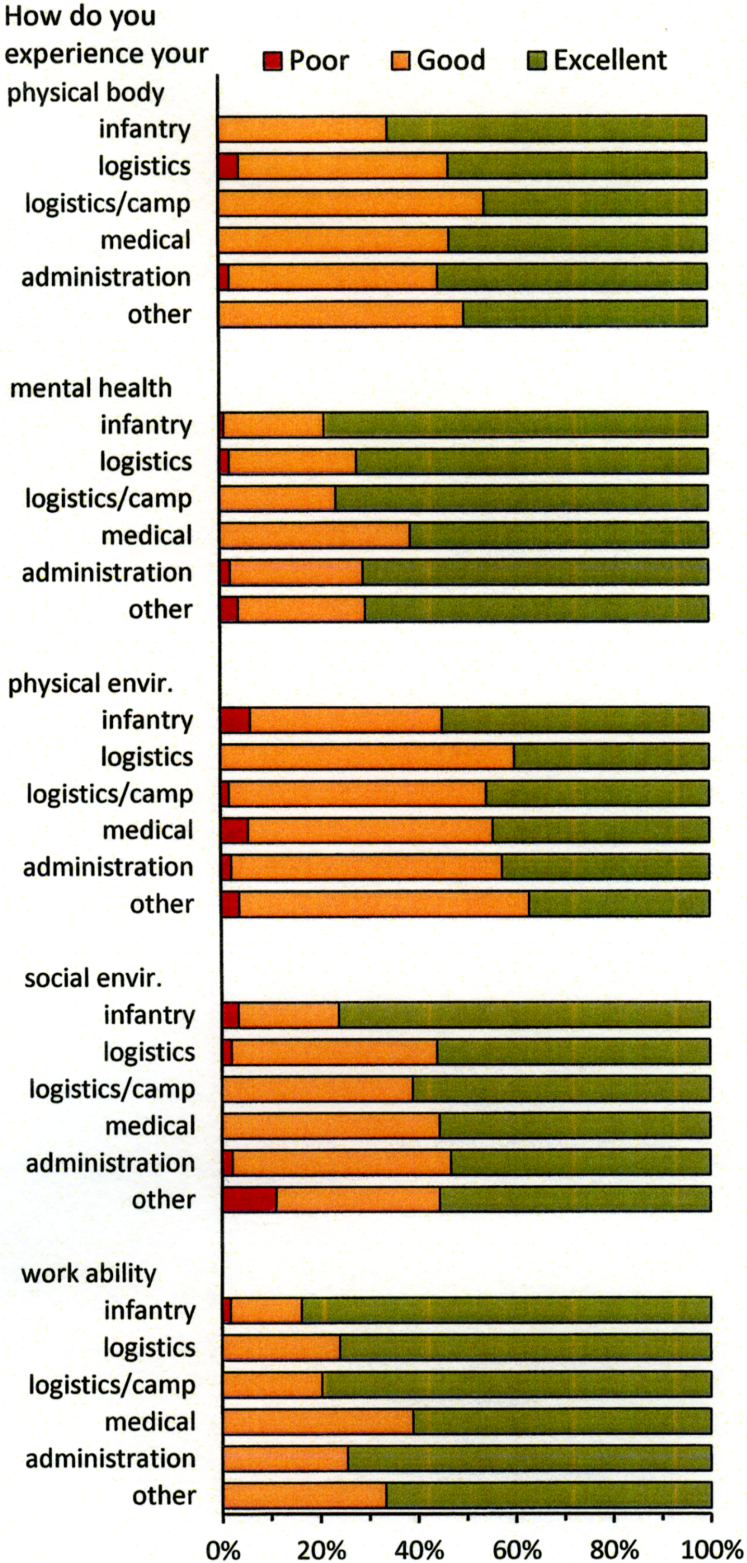
Perceived health of the physical body, mental health, physical environment, social environment and work ability for each military occupational specialty.

Overall, soldiers reported their perceived health in terms of physical body, mental health, physical environment, social environment and work ability as good-to-excellent in all military occupational specialties, Fig 5.

## Discussion

The aim of the study was to describe the prevalence of musculoskeletal complaints and injuries in different Swedish military occupational specialties’ and their estimated exposures to physical workloads in most common tasks during a six-month international deployment in Afghanistan. The present study revealed a high prevalence of MSCI during the mission, which is consistent with previous research by Konitzer et al, who found higher rates of musculoskeletal pain during deployment than before [16]. Also, Glad et al. found that the majority of reported MSCI in Swedish soldiers serving in Afghanistan occurred during deployment [12]. Roy et al. found exacerbations of previous injuries as one of the most common injury causes during deployment [9]. Even though the soldiers seem to be physically well prepared, the increased prevalence of MSCI during deployment might be explained by limited possibilities for recovery, as Rhon found in US soldiers serving in Iraq [17]. Another explanation is that MSCI are training-related and occur during physical training and sports. Roy et al. found weight lifting and sports as common causes of musculoskeletal injuries [9]. The high prevalence of MSCI during deployment indicates a need for physical therapists in areas of operation, with the aim of enhancing early rehabilitation and advising on physical training regimes.

The prevalence of MSCI, of 47%, in the present study is consistent with Roy et al. who reported that 45% of the US soldiers on deployment in Afghanistan sustained an injury [10]. However, the findings are lower when compared to research by Larsson et al. on Swedish conscripts [4]. The prevalence of MSCI in the present study is also lower compared to previous findings by Glad et al. regarding Swedish soldiers serving in Afghanistan [12]. The different results might be explained by various characteristics of the operational units examined, specifically, but not limited to, physical exposures, i.e. time-varying military tasks, climate and military threats in the area of operation. The unit examined might have been exposed to less exhausting physical loads compared to previous Swedish units. Besides different physical exposures during deployment, different definitions of injury might have resulted in discrepancies. In the present study, the questions were on musculoskeletal complaints and injuries, which also include minor disorders. This makes it difficult to compare our findings with research on diagnosed injuries that may include only more serious conditions.

The low prevalence of MSCI at the end of the mission is interesting, since opportunities for rehabilitation during deployment are limited. This might indicate that reported MSCI are of mild character and possible to handle by self-management. However, specifically related to this study, a physical therapist visited the camp for a two-week period to assist with rehabilitation and support.

The findings of lower back and knee as common anatomical locations of MSCI are consistent with previous research [4, 10, 17]. MSCI of the lower back were common in almost all MOS, even though weights of uniforms/equipment carried differed significantly between MOS.

Further causes for the development of MSCI could be prolonged sitting or standing in static and non-ergonomic positions when performing the most common tasks, i.e. soldiers in Infantry and Logistics often travel in an armoured vehicle “RG-32M”. According to reports from physical therapists and ergonomic specialists in SwAF, this vehicle has very poor ergonomic mechanics, which could lead to the development of MSCI. Consistent with the previous assumption, Hauert et al. have found that prolonged periods in static positions are related to overuse injuries [5]. Furthermore, sitting in vibrating vehicles, wearing combat gear during prolonged transportation in non-ergonomic positions, and heavy and frequent carrying of combat loads increase compression load on the lower back. This is consistent with the study by Roy et al, where soldiers in Infantry MOS showed a high prevalence of lower back pain [9]. The findings in the present study show that there is a need for preventive interventions. Soldiers in Infantry and Logistics reported a higher prevalence of MSCI after the mission compared to before. Those two categories of soldiers are exposed to both foot-patrolling and prolonged sitting in poor conditions, i.e. travelling in armoured vehicles. Moreover, they also have limited opportunities for rehabilitation, self-management and recovery compared to other groups, since soldiers in Infantry and Logistics are out in the field and do not stay in camp most of the time.

The most commonly performed tasks that soldiers reported differed between and within the respective MOS, which was also similar with respect to our findings of varying physical workload exposure across military occupational specialties. The soldiers in the administration specialty reported administrative work and staff duties as being their most common tasks. These duties are conducted in camp, and they only wear uniforms and guns while standing or sitting in office-like environments. However, vehicle patrols were reported as another common task by the Administration soldiers, where they were required to wear combat gear, indicating exposure to more exhausting/strenuous duties at times. Logistic soldiers reported transportation outside and between camps as their most common task, but also vehicle patrolling and guard duties. Logistics/camp soldiers perform administrative duties, provide technical support, and maintain equipment and vehicles mostly in camp. For these purposes, they do not wear heavy equipment several times per week or even for several hours per day. However, they often perform their duties, such as vehicle repair or computer work requiring long time periods, in non-ergonomic positions. Soldiers in Infantry reported vehicle patrolling as the most common task, requiring them to wear heavy equipment several times per week lasting from a few hours up to over seventeen hours at a time. During many of the reported tasks, i.e. vehicle patrolling or administrative duties, soldiers are required to remain in prolonged sitting or standing positions. Sitting or standing in non-ergonomic positions in vehicles driving through rough terrain are some of the factors that Hauret et al. relate to overuse injuries [5]. The most common task for medical staff was medical readiness, which is often performed in camp, i.e. at the medical centre in the camp, but could also include tasks out in the field among the Infantry soldiers, i.e. the medical evacuation team. For the military occupational specialty “Other”, soldiers are required to perform tasks both in camp and outside camp, which could lead to different estimations of physical workload exposure.

In the present study, almost half of the soldiers who reported MSCI had their work ability affected because of it. However, only a few rated that the impact of MSCI on work ability was to a great extent, and only 5% were relieved of duties. Other studies have found that up to 28% of the injured soldiers had their duties restricted [9] and could only perform a limited range of tasks during deployment [10], which, inadvertently, affected military readiness [3]. These results are in contrast with findings by Glad et al, who found that the majority of soldiers with musculoskeletal disorders reported low pain/low disabilities which, in most of the cases had no influence on their daily duties [12]. Possible explanations for the different results could be the unique physical challenges of each and every operation and the different operative definitions of MSCI and disability. One could speculate that the low sick-listing rates might be due to high job dedications and loyalties to colleagues, but also due to limited possibilities of sick leave during deployment.

This study revealed that many soldiers report the experience of MSCI which produced a high prevalence rate in this cohort. However, they perceived health of their “physical body” to be good to excellent. One explanation could be that soldiers are used to these conditions, i.e. experiencing MSCI, and have therefore mentally adapted to this being part of their normal work life.

The results pertaining to Infantry soldiers revealed exposure to high physical workloads almost every day of the week with limited opportunities for recovery. Estimated weights of equipment carried agreed with Goff et al, who found 46.3 kg in US soldiers to be the norm [11]. In a study by Roy, it was suggested that duties 6–7 days a week during deployment in Afghanistan might result in overuse injuries [9]. Rhon et al. found the experience of back pain to be associated with soldiers wearing their body armour for several hours a day almost every day without the needed time for recovery [17], indicating that frequent carrying of heavy loads may impact soldiers’ performance. With tasks and occupation-related physical workloads differing within MOS, analysing occupational requirements and developing relevant and fair physical selection-tests should be key requirements [18]. Therefore, during the recruitment process, the individual’s physical capacities should match the physical job requirements and duty-related competences. Moreover, early identification and management of MSCI are important before and during deployment [19], especially when exacerbations of pre-existing injuries are common [10].

Roy et al. found associations between MOS and injury diagnosis, and concluded that injury prevention strategies should focus on risk factors in the soldiers’ job requirements [9]. Concerning the physical characteristics and reported physical loads of the soldiers in Infantry and Logistic MOS, the suggestions by Knapik et al. might be suitable: prevention strategies, improvement of carried equipment’s distribution (close to the body) and lightening of loads carried to reduce injury risks [20]. Zambraski et al. suggest injury prevention strategies such as progressive increased combat loads of the soldiers, especially during the training phase, as well as training of proprioception, balance and agility [2]. Concerning soldiers who reported MSCI of the lower back, primary prevention strategies, as suggested by George et al., should focus on education combined with physical exercises. [21] The education should focus on evidence-based information on lower back pain, particularly focusing on encouraging active coping strategies as well as including physical exercise. It is also important to teach soldiers the principles of self-management and the importance of preventive actions for developing MSCI through regular physical exercise, ensuring sufficient time for recovery after demanding duties, and by maintaining good nutrition.

## Conclusion

Musculoskeletal complaints and injuries during international deployment are common among Swedish soldiers. Furthermore, the soldiers are exposed to duties, which could be highly demanding on their maximum and endurance strength, aerobic and anaerobic endurance, all of which contribute to a high physical workload. The results of the present study indicate the need for the SwAF to further develop strategies focusing on matching the soldiers’ capacity to the job requirements, with relevant and fair physical selection-tests during the recruitment process and proactive interventions targeting MSCI before and during deployment, in order to enhance soldiers’ readiness and promote operational readiness.
